# Context dependent memory in two learning environments: the tutorial room and the operating theatre

**DOI:** 10.1186/1472-6920-13-118

**Published:** 2013-09-01

**Authors:** Andrew P Coveney, Timothy Switzer, Mark A Corrigan, Henry P Redmond

**Affiliations:** 1 Department of Academic Surgery, Cork University Hospital, Cork, Ireland

**Keywords:** Memory, Context-dependent, Learning environment, Free recall, Medical education, Workplace training

## Abstract

**Background:**

Psychologists have previously demonstrated that information recall is context dependent. However, how this influences the way we deliver medical education is unclear. This study aimed to determine if changing the recall context from the learning context affects the ability of medical students to recall information.

**Methods:**

Using a free recall experimental model, fourteen medical student participants were administered audio lists of 30 words in two separate learning environments, a tutorial room and an operating theatre. They were then asked to recall the words in both environments. While in the operating theatre participants wore appropriate surgical clothing and assembled around an operating table. While in the tutorial room, participants dressed casually and were seated around a table. Students experienced the same duration (15 minutes) and disruption in both environments.

**Results:**

The mean recall score from the 28 tests performed in the same environment was 12.96 +/− 3.93 (mean, SD). The mean recall score from the 28 tests performed in an alternative environment to the learning episode was 13.5 +/− 5.31(mean, SD), indicating that changing the recall environment from the learning environment does not cause any statistical difference (p=0.58). The average recall score of participants who learned and recalled in the tutorial room was 13.0 +/− 3.84 (mean, SD). The average recall score of participants who learnt and recalled in the operating theatre was 12.92 +/− 4.18 (mean, SD), representing no significant difference between the two environments for learning (p=0.4792).

**Conclusions:**

The results support the continued use of tutorial rooms and operating theatres as appropriate environments in which to teach medical students, with no significant difference in information recall seen either due to a same context effect or specific context effect.

## Background

The delivery of medical education is moving away from the traditional apprenticeship model, to a model structured on educational initiatives such as simulation and problem based learning [1,2]. This has been motivated by several factors encompassed in Ozuahs concept of the “erosion of the clinical environment” [3]. A move away from in-patient care towards community delivered treatments [4] represents one such challange to the traditional model, rationing a reducing number of available in-patients, to an ever increasing number of undergraduate health care trainees. In Australia for example the number of domestic medical graduates was expected to double between 2005 and 2012 [5]. The often discussed European Working Time directive (EWTD), limiting the working hours of doctors to 48hrs per week from August 1^st^ 2009 has further implications for post graduate training, reducing the amount of clinical experience and training opportunities available to surgical trainees [6]. A similar reduction in working hours in the United States is also raising concerns about the quality of training there [7].

As a result of this gradual erosion in the traditional structures of medical education there has been a shift towards exploring new ways to deliver teaching. We have previously demonstrated that problem based learning and peer to peer undergraduate teaching can be delivered in this way [8]. In a similar fashion it is possible to successfully target specific clinical issues, such as infection control, with targeted e-learning initiatives [9]. Similarly clinical simulation has also been shown to improve post graduate training [10], providing a safe, supportive educational environment [11].

Fundemental to many of these initiatives is the recreation of specific clinical environments, either virtually or through use of specific simulators. However, due to the aforementioned factors, much of the formal teaching we now deliver is based outside of the clinical environment in tutorial rooms and lecture halls. This is despite evidence that distinct environments offer differing contexts for learning, and so materially influence the ability of subjects to recall data. One such study [12] utilised the two very distinct environments of underwater and land to demonstrate this phenomenon. Using a free recall experimental model, participants demonstrated a superior ability to recall data while in the same environment in which they were originally taught. This is the “same context” effect.

A subsequent paper by Fernandez and Glenberg using eight separate experiments contradicted these findings and argued that changing environment context does not reliably effect memory [13]. More recently in a more clinically relevant study by Koens et al. [14], the classroom and bedside were used as two learning and recall environments, where both random words as well as a clinical case were used as the subject matter. The influence of changing the recall context from the learning context did not change the participants’ recall of the random material nor the context relevant clinical case material. This would suggest that the relevance of the learning context to the subject matter being learned might not be as influential as expected.

Finn et al. demonstrated that medical student clothing had a ‘moderate’ effect on data recall [15]. In this study, medical students separated into two equivalent groups and learned renal anatomy in either “scrubs” or casual clothing and then were tested five weeks later in their causal clothing. The students who wore casual clothing performed better in their recall test of renal anatomy compared to those that wore “scrubs” at the time of teaching. The authors hypothesised that the change in clothes represented a change between the learning and recall context and negatively impacted on student recall.

A meta-analysis of 75 studies by Smith et al. [16] examining the effects of context on memory, found that across multiple studies, environmental context effects were reliable. Their meta-analysis was based on four primary hypotheses: “reinstatment”, “outshining”, “overshadowing” and “mental reinstatment”. They concluded that environmental context-dependent memory effects are less likely to occur under conditions in which the immediate environment is likely to be suppressed. This suppression of the immediate environment may be intentional, such as when subjects avert their gaze or even close their eyes when trying to facilitate conceptual taught, by disengagement from processing their immediate environmental surroundings, freeing up cognitive resources. The suppression of the immediate environment may also be non-intentional when the activity or task being performed by the subject requires such concentration and attention that it “overshaows” the environmental cues in the learning environment or “outshines” any environmental cues if it occurs during retrieval.

The operating theatre represents a unique learning environment for medical students and surgical trainees. It is unique on a multisensory level, visually (sterile cold environment), auditory (alarms, monitors, shouting), olfactory (cautery, feaces, pus), tactile (wearing scrubs, cap, facemask) and also often represents a pressurised dynamic environment. Therefore, suppression of the operating environment is very difficult, which would indicate that learning in such an environment is susceptible to context-dependent memory effects. Historically, surgical trainees spend tens of thousands of hours training in the operating theatre environment before becoming a consultant. It is therefore very relevant that such an environment should be optimised to facilitate learning and recall.

There are two aims of this study. The first aim of the study is to determine if there is a “same context effect” by determining if changing the recall environment from the learning environment negatively impacts subject’s recall performance. The second aim is to determine if there is a “specifc context effect” by comparing the recall scores of students learning in either the operating theatre or a tutorial room environment.

## Methods

### Subjects

In total, 14 third and fourth year undergraduate medical student volunteers were recruited by email to participate in the study. All participants were fluent in English, with an even distribution of gender, ethinicity and age profile consistent with a contempary multinational medical undergraduate class. The identity of participants was annonymised. Ethical approval was granted by the Clinical Research Ethics Committee of Cork University teaching hospitals. Due to limited space in the operating room, a total of 16 participants were recruited. Unfortunately, two volunteers failed to attend at short notice on the research day, resulting in 14 participants.

### Environments

A tutorial room, a coffee room and an operating theatre were used as three separate environments in this study. Participants were required to dress into appropriate surgical attire before entering the operating theatre. This included “surgical scrubs”, shoe covers, a theatre cap and a face mask. While in the tutorial room and coffee room participants wore casual cloths. Participants remained standing in the operating theatre but were seated in the tutorial room. It has been demonstrated that the difference in recall when the recall environment is changed relative to the learning environment may actually be due to the disruption experienced when changing from one environment to another rather then the actual change in environments [17]. To control for this all participants were required to leave their learning envrionment to a coffee room before returning to their recall environment. This ensured that all participants experienced the same level of distrubition during their 15-minute retention interval regardless to whether they were changing environments or not. Light refreshments were provided in the coffee room.

### Learning material (word lists)

In order to control for differing medical knowledge, four lists of non medical words (List 1–4), each consisting of 30 unrelated, different one-, two- and three-syllable nouns randomly chosen by a free online random word generator [18] were constructed and recorded as audio files (Additional file 1). The same audio files were used for each participant to ensure consistency. The audio file started with instructions for the participants and after a five second warning the list of 30 words started in sequence with one word given every two seconds. The list was repeated after a ten second interval, giving participants two attempts in total to listen to and memorise the list. On completion, the participants left their learning environment, changed clothes if needed, and returned to the coffee room for a 15 minute break. Discussion or verbal rehersal of the word list was not permitted during this time.

### Recall assessment

After a fifteen-minute break in the coffee room participants returned to a designated environment (operating room or tutorial room). Their ability to recall their list of 30 words was assessed by asking them to write down as many words as they could remember on a blank sheet of paper in five minutes. This activity was performed while standing holding a clip board in the operating theatre, or sitting down while in the tutorial room. Correct spelling of the words was not assessed.

### Procedure

**Figure 1:**
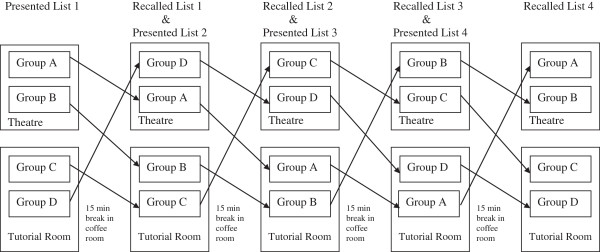
Flow chart of groups and order of learning environments used for learning and recalling word lists 1–4.

The fourteen subjects were divided into four groups A, B, C and D in the coffee room. As depicted in Figure 1, Groups A and B were sent to the Operating Theatre to listen to List 1. Groups C and D were sent to the Tutorial room to listen to List 1. All groups then returned to the coffee room for a fifteen minute break. Groups A and D then returned to the Operating theatre and Groups B and C returned to the Tutorial room and assessment of recall was performed. The groups then remained in their environments and after a short five minute break all groups were presented with List 2 to recall. All groups returned again to the coffee room for another 15 minute break. The groups then returned to their appropriate rooms to assess their recall of List 2. List 3 and List 4 were presented to the groups in their appropriate environments in a similar manner ensuring that each of the four groups experienced the four different combinations of learning/recall environments listed below.

OT / OT – Learned in the Operating Theatre and Recalled in the Operating Theatre

OT / TR – Learned in the Operating Theatre and Recalled in the Tutorial Room

TR / TR – Learned in the Tutorial Room and Recalled in the Tutorial Room

TR / OT – Learned in the Tutorial Room and Recalled in the Operating Theatre.

Each list lasted approximately 3 min 30secs. There was a fifteen minute break between the learning and recall sessions. Each recall session lasted five minutes and was followed by a five minute break before starting the next learning session. Therefore each of the four environment combinations lasted 28.5 minutes meaning that the four combinations made up a total experiment time of approximately two hours, which was performed from 8 – 10 pm when theatre activity was quiet.

### Statistics

The effects of changing the learning and recall environments on recall performance was assessed by comparing recall scores for the different combinations of environments using a Wilcoxon matched-pairs, signed-ranks test to test for significance.

## Results

Fourteen volunteers participated, undergoing 4 individual memory tests using each of the four possible environment combinations. This gave a total of 56 recall scores for analysis. The median recall score from all memory tests was 13.5/30 with an interquartile range of 6.25. The interquartile range test for normality of distribution confirms a Gaussian distribution of the recall scores.

The results of participants’ recall scores are displayed (Table 1). The mean recall score from the 28 tests performed in the same environment to the learning episode was 12.96 +/− 3.93 (mean, SD). The mean recall score from the 28 tests performed in an alternative environment to the learning episode was 13.5 +/− 5.31(mean, SD), indicating that changing the recall environment from the learning environment does not cause a statistically significant difference (p=0.581).

**Table 1:** Individual recall scores for each of the 14 subjects A1-D3, for each of the learning and recall environment combinations, where “OT / TR” represents learnt in the operating theatre and recalled in the tutorial room

**Subject**	**TR / TR**	**OT / OT**	**OT / TR**	**TR / OT**	**Means**
A1	17	14	22	22	18.75
A2	18	8	12	13	12.75
A3	6	13	12	4	8.75
A4	15	18	19	11	15.75
B1	11	8	9	10	9.5
B2	14	9	12	14	12.25
B3	6	9	9	8	8
C1	10	9	4	6	7.25
C2	14	12	15	14	13.75
C3	13	14	19	14	15
C4	11	16	15	17	14.75
D1	15	21	26	16	19.5
D2	14	18	17	10	14.75
D3	18	12	17	11	14.5

(Means range 7.25 – 19.5).

Changing the recall environment from the learning environment, increased the recall score for seven participants, reduced the recall score for six participants and had no effect on the recall score for one participant (Table 2).

**Table 2:** Averaged recall scores for subjects A1-D3 according to whether the recall environment was the same or changed from the learning environment

**Subjects**	**Same environment**	**Changed environment**
A1	15.5	22
A2	13	12.5
A3	9.5	8
A4	16.5	15
B1	9.5	9.5
B2	11.5	13
B3	7.5	8.5
C1	9.5	5
C2	13	14.5
C3	13.5	16.5
C4	13.5	16
D1	18	21
D2	16	13.5
D3	15	14
Means	12.96	13.50

The average recall score of participants who learned and recalled in the tutorial room was 13.0 +/− 3.84 (mean, SD). The average recall score of participants who learned and recalled in the operating theatre was 12.92 +/− 4.16 (mean, SD), representing no significant difference between the two environments for learning (p=0.96). When the recall scores of subjects who learned lists in the operating theatre were compared to the recall scores of subjects who learned lists in the tutorial room were compared, (regardless to where the recall test was performed) no significant difference was seen.

The mean recall scores for the four word lists were 12.0, 14.1, 14.2, 12.6 respectively. The four word lists used for recall tests appear to be of similar difficulty as no statistical difference was seen in recall scores for the different word lists. (p=0.12 for list1 compared to list3).

## Discussion

Clinical limitations may mean that the delivery of medical teaching shifts to other settings such as clinically simulated environments, or clinically distinct environments such as tutorial rooms. This study was designed to determine if the use of such distinct environments had any effect on student data recall. Using the free recall model of Godden & Baddeley [12] in the two contrasting environments of a surgical theatre and a tutorial room, we failed to show any significant difference in recall ability of participants due to variation in learning or recall environments indicating that a specific context effect was not evident. Similarly, changing the recall environment from the learning environment did not lead to any significant change in recall scores, indicating that “same context effect” was not evident.

Our findings are similar to the findings of Koens et al. [14]. Like the Koens study, we compared the influence of a clinical (operating theatre) and educational (tutorial room) teaching environment on subjects’ recall scores of random word lists. As in the Koens study, no combination of teaching and recall environments altered recall scores significantly. They used a similar number of recall scores from each environment combination for comparison as was used in our study. However our study differed, in that we used the same students in each of the four environmental combinations so that inherent differences in student’s innate recall ability would not influence their recall scores. Using the same students in each of the four different environment combinations necessitated using four separate word lists. The content of the word lists didn’t appear to influence recall scores, as there was no significant difference seen in the recall scores of any one list compared to the other three.

Two potential explanations exist for the differing results between this study and that of Godden & Baddeley. Firstly the difference between a tutorial room and an operating theatre is not as obvious as that of dry land and underwater. However if we are to maximise the positive influence of teaching environments on learning, even subtle differences between environments should be critically explored. Using the two realistic learning environments of a tutorial room and operating theatre, while not as different as underwater and dry land, are far more relevant to surgical education and make our study findings more applicable and valid to surgical teaching practice.

Secondly there is a distinct physiological difference between those on land and those underwater. This is clearly not the case between an operating theatre and a tutorial room, although subtle differences may exist, they were not the focus of this study. Divers experience physiological changes underwater, like being weightless and being restricted of sight. One study [19] found that students, who recalled words in the same physiological state (i.e., with regulated heartbeats) as where they learned them, performed significantly better than students who experienced opposite states. This may be of particular relevance to surgical training, as surgeons are often forced to perform under pressure producing adrenaline and a corresponding physiological stress response. This physiological state, although not measured, was not replicated in our simulated surgical environment and therefore represents a limitation to our study.

There are some other limitations to our study, which should be acknowledged. A relatively short-term memory was examined, with a retention interval of just 15 minutes separating the learning and recall sessions. As learning in medical education requires both short-term and long-term memory, the findings of our study may be less applicable. Our reasoning for assessing 15-min interval memory in our study was that the recall scores would be higher and more likely to demonstrate a larger difference, if one existed than if we used a longer interval period. Logistically a shorter interval period was considered too challenging, as time was required to facilitate a change of learning environments, which necessitated changing into and out of surgical scrubs. The effect of duration of retention interval examined by Smith & Vela’s meta-analysis [16] didn’t show a significant difference in context effect between recall at 5 minutes up to 1 day, justifying our use of a 15-minute retention interval.

Random word lists, which represent non-meaningful subject matter were deliberately selected, so that inter-participant variation in medical knowledge would not influence participants’ recall scores. These random word lists obviously had no relevance to the learning environments in which they were being learnt. This is not representative of the real clinical learning environment, where the learning content will be relevant to the clinical environment. Fernandez & Glenberg [13] proposed that studies of the effect of environment on memory are likely to be more valid if “they deal with memory for events that are perceived to be related to the environment”. This lack of relationship between our word list and our learning environment may explain our negative results. The use of surgically relevant material such as anatomy or surgical technique might yield different results.

Another potential criticism of the study is the effect of participants having to learn and recall four separate word lists in a row. The reasoning behind this was to facilitate a within-subjects study, where participants act as their own comparison, thereby negating inevitable variation in inherent recall ability among participants. Learning and recalling four separate word lists does have the potential to introduce unwanted influences on results such as improved recall performance due to “practice effect” or impaired recall performance due to fatigue. A study by Falleti et al. [20], examined for “Practice effects” associated with repeated cognitive tests using four repetitions with 10-minute test-retest intervals similar to our study and found that such an effect existed between the first and second test but did not for subsequent tests. A significant variation in recall performance according to the order of our word lists was not seen. There was no statistical difference seen between the average recall scores of any of the four word lists. This would also support that the lists were equivalent in their level of difficulty to recall which was expected from the random method in which they were derived. As Falleti also alluded to, it is possible that the beneficial effects of practice were counter-balanced by participant fatigue resulting in a consistent recall score across the repeated recall tests. This is unlikely though. While fatigue was not objectively measured, participants were in good spirits throughout the entire study period, even before the final recall test. The recall of four word lists consecutively could potentially lead to confusion and “recall intrusion”, where words from previous lists were recalled on to the wrong list. This however was only seen once throughout all the recall tests. The fact that participants were aware that they were under scrutiny, increased their motivation to perform, consistent with the Hawthorne effect [21].

During each of the simulated learning environments, participants were only presented with auditory information, in the form of a pre-recorded word list. In practice during a learning session in the operating theatre or tutorial room, students will be presented with a significant amount of visual information also which will impact on their overall recall. One encoding strategy used by some participants was to close their eyes, thereby excluding any visual influence or distraction and allowing complete concentration on the audio list. This might also have contributed to the lack of influence of environment on recall scores in this study. As similar “remembering behaviour” by subjects has been described by Glenberg et al. [22], where subjects avert their gaze from their immediate experimental surroundings to facilitate disengagement from their environment, resulting in better recall.

Having demonstrated that there is no significant difference in short term recall between the two environments, it is worth exploring the advantages to teaching in a controlled environment such as a tutorial room. A non-clinical setting, allows teachers and students to focus on pre-defined essential learning outcomes rather than allowing the heterogeneity of clinical cases to determine the learning outcomes of a teaching session. Teaching in the clinical environment is often opportunistic and therefore there can be significant variation in the clinical exposure students experience [23]. With students and trainees attached to increasingly subspecialised teams within medicine and surgery, they may fail to experience some of the basic general clinical cases that form an essential part of an undergraduate and postgraduate medical education, which may be of particular importance in providing an out of hours emergency service [24]. Delivery of teaching of core curriculum in the pre-planned non-clinical setting in parallel with ongoing clinical attachments ensures that basic core topics, which may be missed in the clinical setting, are covered. A controlled tutorial setting also facilitates consistent homogenous delivery of content, which is imperative when standardizing content across larger class sizes. The definition and standard delivery of a core undergraduate medical curriculum was one of the key recommendations by the General Medical Council, signaling an end to the tradition of purely opportunistic teaching in which students’ experiences can vary widely [25].

## Conclusions

This study has demonstrated that changing environments between an operating theatre and a tutorial room does not significantly affect the short-term recall ability of participants. The use of non-clinical environments such as tutorial rooms or lecture theatres to deliver medical education should continue to be used to teach students.

## Competing interests

The authors declare that they have no competing interests.

## Authors’ contributions

AC participated in study design, running of the study, data collection and analysis and writing of article. TS participated in study design, running of the study and data collection. MC participated in study design and writing of the article. HR participated in the study design and writing of the article. All authors read and approved the final manuscript.

## Pre-publication history

The pre-publication history for this paper can be accessed here:

http://www.biomedcentral.com/1472-6920/13/118/prepub

## Supplementary Material

Additional file 1 Lists of words recorded on audio files. Click here for file
