# Supplementary material for: Context dependent memory in two learning environments: the tutorial room and the operating theatre
Source: BMC Med Educ. 2013 Sep 1;13:118. doi: 10.1186/1472-6920-13-118 (PMC3766034; doi:10.1186/1472-6920-13-118)
Supplement: Additional file 1 — Lists of words recorded on audio files. [file 1472-6920-13-118-S1.doc]

**Additional file 1 – Lists of words recorded on audio files.**

**LIST 1**

Syndrome

Code

Revision

Van

Mother

Cloth

Committee

Forecast

Choice

Diary

Function

Bird

Painting

Elevator

Brass

Killer

Bear

Panel

Random

Fault

Finger

Track

Stand

Budget

Customer

Week

Delight

Alarm

Rubber

Car

**LIST 2**

Helmet

Head

Controller

Boat

Fire

Pig

Supporter

Actor

Leaf

Insurance

Treatment

Fork

Morning

Illusion

Book

Bottle

Nail

Study

Dentist

Hole

Excuse

Strike

Pay

Chamber

Discipline

Mark

Service

Inside

Female

Drug

**LIST 3**

Season

Sun

Departure

Ball

River

Breeze

Uniform

Colon

Script

Policy

Verdict

Pork

Wedding

Digital

Cloud

Pilot

Mob

Carpet

Junction

Flight

City

Prince

Dog

Filter

Manager

Art

Power

Income

Program

Press

**LIST 4**

Tactic

Knife

Mixture

Nut

Journey

Rose

Episode

Chemist

Grasp

Disorder

Copper Hook

Railway

Document

Team

Honey

Gate

Servant

Fragment

Mouse

Creature

Cross

Gun

Future

Disturbance

Rat

Counter

Report

Food

Lock
